# The Association Between Self-Reported Hearing Loss and Loss of Usual Source of Health Care Among Older Medicare Beneficiaries: Evidence From the National Health and Aging Trends Study

**DOI:** 10.1093/geroni/igad002

**Published:** 2023-01-16

**Authors:** Emmanuel Garcia Morales, Lama Assi, Danielle Powell, Kayti Luu, Nicholas Reed

**Affiliations:** Cochlear Center for Hearing and Public Health, Johns Hopkins Bloomberg School of Public Health, Baltimore, Maryland, USA; Department of Ophthalmology, Louisiana State University Health Sciences Center, New Orleans, Louisiana, USA; Department of Health Policy and Management, Johns Hopkins Bloomberg School of Public Health, Baltimore, Maryland, USA; John A. Burns School of Medicine, University of Hawai’i at Mānoa, Honolulu, Hawai’i, USA; Cochlear Center for Hearing and Public Health, Johns Hopkins Bloomberg School of Public Health, Baltimore, Maryland, USA; Department of Epidemiology, Johns Hopkins Bloomberg School of Public Health, Baltimore, Maryland, USA

**Keywords:** Hearing aids, Hearing loss, Usual source of care

## Abstract

**Background and Objectives:**

The purpose of the study is to investigate the association of hearing loss (HL) with maintaining a usual source of care (USOC).

**Research Design and Methods:**

In this study we implemented a time-to-event analysis using data from the National Health and Aging Trends Study (NHATS), a nationally representative study of older Medicare beneficiaries in the United States. The study sample included 2 114 older adults, aged 65+ years, 58.9% female, 20.4% Black, who reported having a USOC during the baseline round of NHATS and who remained community-dwelling during the 2011–2018 study period. Based on self-report measures at baseline, individuals’ hearing status was classified into 3 categories: no HL, treated HL (hearing aids users), and untreated HL (nonhearing aid users who reported having hearing difficulties). Time-to-event was computed as the time elapsed between baseline and the study round in which the respondent first reported no longer having a USOC. Discrete-time proportional hazard models were estimated.

**Results:**

In fully adjusted models, untreated HL at baseline was associated with a hazard ratio (HR) for losing one’s USOC 1.60 (95% confidence interval: 1.01, 2.56) times higher than that of participants with no HL. We found no HR differences between the treated- and no-HL group.

**Discussion and Implications:**

Untreated HL at baseline was associated with a higher probability of losing one’s USOC over time. Noninvasive interventions such as hearing aids may be beneficial for maintaining a USOC.

**Translational Significance:** Having a usual source of care (USOC) has been associated with health care access, quality of health services, and better health; however, individuals with hearing loss (HL) are less likely to report having a USOC than those without HL. In this research, we found that untreated HL at baseline is associated with a higher risk of reporting losing one’s USOC. We estimated no differences in the risk of losing a USOC between adults with treated HL (hearing aid users) and individuals without HL. Noninvasive interventions such as hearing aids may be a protective factor for maintaining a USOC.

Having a usual source of care (USOC) has been associated with improved health care access, quality of services, and health overall (1,2). Previous research suggests that patients with a USOC are more likely to seek timely care, receive appropriate treatment management, especially for chronic conditions (3,4), and engage in preventive health services (5,6). Given the importance of maintaining a USOC for health outcomes and potential savings from efficient use of health care services (7,8), understanding risk factors associated with not having a USOC would provide useful evidence for policymakers and other health care stakeholders.

In the United States, about 95% of older Medicare beneficiaries have access to a USOC (9). Despite this high proportion, potentially modifiable risk factors for the loss of USOC among this group include: transportation difficulties, insurance coverage, and depression (10). Moreover, recent work has pointed at sensory loss (including hearing loss [HL]) as an additional risk factor associated with a lack of USOC (11).

HL is strongly associated with age, affects nearly two thirds of U.S. adults aged 70+ years (12), and is projected to affect more than 70 million older Americans by 2060 (13). Given the importance of patient–provider communication for health literacy (14) and treatment adherence (15), unsuccessful patient–provider communication has been suggested as a potential mediator for the association between HL, unmet health care needs, dissatisfaction with care (16), and higher health care utilization and expenditures (17). In addition, communication barriers could influence someone’s interactions with health providers (18), thus explaining previous findings that older adults with HL are more likely to lack a USOC than those who reported no difficulty hearing (16).

Prior work regarding the association between HL and USOC among older adults has produced heterogeneous results. One study in the United States found that after controlling for patient characteristics, self-reported HL was not associated with having a USOC (11). Another study reported that individuals with the highest degree of self-report hearing difficulties were more likely to report lacking a USOC (16). Both studies were cross-sectional, and no distinction between treated and untreated HL was analyzed. In this study, we use longitudinal data from the National Health and Aging Trends Study (NHATS) to examine the association between treated and untreated HL at baseline, with loss of USOC among community-dwelling older adults over an 8-year period.

## Method

### Study Population

We used data from the 2011–2018 cycles of the NHATS (19), an ongoing nationally representative, longitudinal cohort study of community-dwelling Medicare beneficiaries aged 65 and older. Study participants were interviewed every year.

Our analytic sample included 2 133 participants who reported having a USOC at baseline, and who were observed and remained community-dwelling during all eight cycles of the study period. Participants in residential care facilities were excluded as they were assumed to have a USOC while in a facility. Participants without a complete set of covariates (*N* = 23) were further excluded, yielding an analytic sample of 2 110 individuals.

### Usual Source of Care

During each interview, participants were asked if “there is a doctor you think of as your regular doctor, that is, a doctor you usually go to when you are sick, and need advice about your health”? Participants responding “yes” were considered as having a USOC. We computed the number of years between baseline and either the first time a participant responded no longer having a USOC (our failure outcome), or missing information about USOC was recorded (treated as right-censoring).

### Hearing Loss

Participants were grouped as: no HL, treated HL, and untreated HL according to baseline provided information. Participants who reported using a hearing aid/device during the month preceding the study interview were categorized as having treated HL. Participants who reported not being able to hear well enough to use the telephone, or carry a conversation with the radio or TV on, were assigned to the untreated-HL group. All other participants were treated as having no HL.

### Covariates

Covariate selection was guided by previous literature on sociodemographic and health characteristics associated with having a USOC or HL (10,11). These include baseline, age, race/ethnicity (White, Black, Hispanic, and other), sex, marital status (married/living with partner, and single/never married/divorced/widow), education (less than high school, high school diploma or equivalent, and some college or more), household income (under the poverty line, 100%–199% the poverty line, and ≥200% of the poverty line), number of chronic health conditions among heart attack, heart disease, high blood pressure, arthritis, osteoporosis, diabetes, lung disease, stroke, or cancer (0, 1–2, 3–5, or 6+), self-reported health status (Likert scale, 1 = Excellent, …, 5 = Poor), number of activities of daily living (ADLs) for which the respondent reported needing help (none, 1–2 ADLs, and 3≤ ADLs), dementia (probable, possible, and no dementia) (20), additional health coverage (Medigap/Medicare supplement, Medicaid, or Tricare), and depression status (based on Patient Health Questionnaire-2 scores ≥3) (21).

Despite being identified as a risk factor for loss of USOC, experiencing transportation barriers (reporting that a transportation problem restricted any activity participation in the month before the interview) was not included in the main analyses due to data availability, as a total of *N* = 1 804 participants had missing information.

### Statistical Analysis

We described differences in baseline demographics between our analytic sample and excluded individuals, and by hearing group using χ^2^ (categorical variables) and Kruskal–Wallis analysis of variance (continuous variables). Given the discrete nature of our data we estimated a discrete-time proportional hazard model using a cloglog model to estimate hazard ratios (HRs) (22) and 95% confidence intervals (CIs) for losing USOC. We verified the proportional hazard assumption by looking at the correlation between Schoenfeld residuals and survival times.

We performed unweighted and weighted (using 2011 enrollment weights) estimations to account for NHATS’ complex survey design. All main analyses were adjusted for participant’s baseline characteristics including: age, sex, race/ethnicity, marital status, education, household income, self-reported health, number of chronic conditions, dementia status, additional health coverage, and depression. All covariates were treated as time-invariant in our analyses. Survival functions by hearing group were estimated for the unweighted unadjusted model.

In secondary analyses, we explored the potential moderation effect of (i) depression and (ii) transportation barriers on the association between hearing groups and self-reported loss of USOC. These secondary analyses were driven by previous findings showing the importance of unmet transportation needs and depression as risk factors for loss of USOC. For the case of analyses pertaining to transportation barriers, a sample of *N* = 320 was used due to missing data.

Finally, as a sensitivity analysis we estimated our main model including all study participants who satisfied our inclusion criteria (community-dwelling, having a USOC, full set of covariates), but who were lost to follow-up during the study period. As participants in residential care facilities were assumed to have a USOC, we excluded at-risk participants who transitioned into residential care from this analysis. All our estimations were performed using Stata/SE 17.0.

## Results

Compared to our analytic sample, excluded individuals were older (79.1 vs 74.5 years), more likely to have less than a high school education (29.1% vs 20.7%), and with a higher comorbidity count (14.7% vs 8.5% with 5+ comorbidities; Supplementary Table S1). Among the 2 110 participants in our sample, 1 721 (81.6%) were assigned to the no-HL group, 155 (7.3%) to the treated-, and 234 (11.1%) to the untreated-HL group. A total of 1 859 (88.1%) participants maintained a USOC, 246 (11.7%) reported losing their USOC at some point, and 5 (0.2%) were right-censored. Compared to the other two groups (no HL, 11.1%; hearing aid users, 11%), a higher proportion of individuals with untreated HL reported losing their USOC (16.2%) (Table 1).

**Table 1.** Baseline Characteristics of the Study Sample by Hearing Loss Categories, National Health and Aging Trends Study (NHATS; 2011–2018)

Characteristic	Total	No Hearing Loss	Treated Hearing Loss	Untreated Hearing Loss	*p* Value
	*N* = 2 110	*n* = 1 721	*n* = 155	*n* = 234	
Usual source of care, *n* (%)					.068
Lost usual source of care	246 (11.7%)	191 (11.1%)	17 (11.0%)	38 (16.2%)	
Kept usual source of care	1 859 (88.1%)	1 525 (88.6%)	138 (89.0%)	196 (83.8%)	
Age (years), median (IQI)	74 (69–79)	73 (69–78)	78 (73–83)	76 (71–82)	<.001
Female, *n* (%)	1 243 (58.9%)	1 075 (62.5%)	57 (36.8%)	111 (47.4%)	<.001
Race/ethnicity, *n* (%)					<.001
White	1 514 (71.8%)	1 199 (69.7%)	137 (88.4%)	178 (76.1%)	
Black	430 (20.4%)	386 (22.4%)	**	34 (14.5%)	
Hispanic/other	166 (7.9%)	136 (7.9%)	**	22 (9.4%)	
Married, *n* (%)	1 223 (58.0%)	979 (56.9%)	108 (69.7%)	136 (58.1%)	.008
Education level, *n* (%)					<.001
Less than high school	436 (20.7%)	351 (20.4%)	18 (11.6%)	67 (28.6%)	
High school diploma or equivalent	539 (25.5%)	438 (25.5%)	40 (25.8%)	61 (26.1%)	
Some college or more	1 135 (53.8%)	932 (54.2%)	97 (62.6%)	106 (45.3%)	
Household income, *n* (%)					<.001
Under poverty line	356 (16.9%)	290 (16.9%)	12 (7.7%)	54 (23.1%)	
100%–199% poverty line	494 (23.4%)	399 (23.2%)	30 (19.4%)	65 (27.8%)	
200%< poverty line	1 260 (59.7%)	1 032 (60.0%)	113 (72.9%)	115 (49.1%)	
Number of comorbidities, *n* (%)					.064
No comorbidities	199 (9.4%)	166 (9.6%)	13 (8.4%)	20 (8.5%)	
1–2 comorbidities	1 001 (47.4%)	836 (48.6%)	72 (46.5%)	93 (39.7%)	
3–4 comorbidities	730 (34.6%)	580 (33.7%)	59 (38.1%)	91 (38.9%)	
5+ comorbidities	180 (8.5%)	139 (8.1%)	11 (7.1%)	30 (12.8%)	
Self-reported health status, *n* (%)					<.001
Poor/fair	394 (18.7%)	321 (18.7%)	15 (9.7%)	58 (24.8%)	
Good	679 (32.2%)	537 (31.2%)	55 (35.5%)	87 (37.2%)	
Very good	692 (32.8%)	569 (33.1%)	63 (40.6%)	60 (25.6%)	
Excellent	345 (1 6.4%)	294 (17.1%)	22 (14.2%)	29 (12.4%)	
Help needed with ADLs, *n* (%)					<.001
No help needed	1 526 (72.3%)	1 262 (73.3%)	122 (78.7%)	142 (60.7%)	
1–2 ADLs	419 (19.9%)	330 (19.2%)	**	61 (26.1%)	
3≤ ADLs	165 (7.8%)	129 (7.5%)	**	31 (13.2%)	
Dementia class, *n* (%)					<.001
Probable dementia	71 (3.4%)	51 (3.0%)	**	16 (6.8%)	
Possible dementia	185 (8.8%)	135 (7.8%)	**	33 (14.1%)	
No dementia	1 854 (87.9%)	1 535 (89.2%)	134 (86.5%)	185 (79.1%)	
Additional health care coverage, *n* (%)					.012
None	632 (30.0%)	530 (30.8%)	32 (20.6%)	70 (29.9%)	
Medigap	1 115 (52.8%)	890 (51.7%)	103 (66.5%)	122 (52.1%)	
Medicaid	238 (11.3%)	196 (11.4%)	10 (6.5%)	32 (13.7%)	
Tricare	125 (5.9%)	105 (6.1%)	10 (6.5%)	10 (4.3%)	
Depression, *n* (%)	227 (10.8%)	178 (10.3%)	**	41 (17.5%)	<.001

*Notes*: ADLs = activities of daily living, IQI = interquartile interval.

**Indicates no data are shown as these cells do not satisfy the minimum cell size requirements imposed by NHATS.

**Figure 1.**
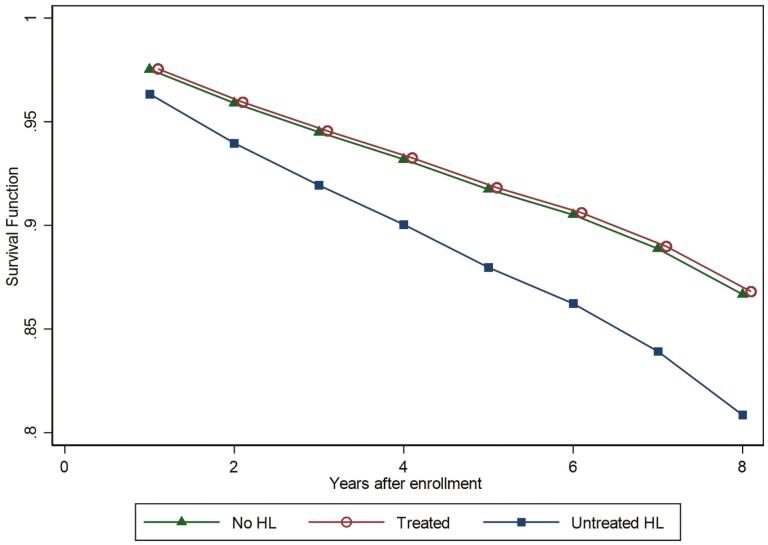
Estimated survival functions for keeping usual care by hearing loss. *Note*: Estimated survival functions were obtained after the unweighted unadjusted model as follows: ${\hat{S}}_{t}=\text{exp}\left(\sum_{s=1}^{t}\text{ln}(1-{\hat{h}}_{s})\right)$.

The estimated survival functions for the no- and treated-HL groups are similar, while the survival function for the untreated group is always under these two curves (see Figure 1). In weighted models, we found that self-reported untreated HL at baseline was associated with increased risk of losing one’s USOC. Participants with untreated HL had a risk for losing one’s USOC 1.60 times (95% CI: 1.01, 2.56) higher than participants with no HL. We found no difference in the risk of losing one’s USOC between hearing aid users and older adults without HL. In contrast, according to our estimates, higher comorbidity count and having additional insurance coverage (Medigap) are both associated with lower risk for losing one’s USOC (Table 2).

**Table 2.** Discrete-Time Proportional Hazard Models for Losing One’s Source of Usual Care and Hearing Loss at Baseline, National Health and Aging Trends Study (*N* = 2 110)

Characteristic	Unweighted		Weighted	
	HR (95% CI)	*p* Value	HR (95% CI)	*p* Value
Hearing				
No hearing loss	REF		REF	
Hearing aid use	1.16 (0.69, 1.94)	.583	0.97 (0.57, 1.65)	.916
Untreated hearing loss	1.52 (1.05, 2.22)	.029	1.6 (1.01, 2.54)	.046
Age	1 (0.98, 1.02)	.748	0.99 (0.97, 1.02)	.592
Female	0.92 (0.69, 1.23)	.588	0.99 (0.71, 1.37)	.936
Race/ethnicity				
White	REF		REF	
Black	1.34 (0.94, 1.90)	.102	1.25 (0.89, 1.76)	.201
Hispanic/other	1.43 (0.92, 2.23)	.11	1.36 (0.86, 2.14)	.183
Married	1.03 (0.75, 1.40)	.872	0.92 (0.64, 1.33)	.661
Education level				
Less than high school	REF		REF	
High school diploma or equivalent	0.95 (0.64, 1.40)	.78	0.91 (0.60, 1.36)	.624
Some college or more	0.77 (0.52, 1.14)	.198	0.83 (0.58, 1.18)	.286
Household income				
Under poverty line	REF		REF	
100%–199% poverty line	0.91 (0.61, 1.35)	.642	0.93 (0.57, 1.52)	.764
200%< poverty line	0.87 (0.56, 1.36)	.552	0.86 (0.47, 1.55)	.607
Help needed with ADLs				
No help needed	REF			
1–2 ADLs	0.97 (0.68, 1.37)	.864	1.03 (0.73, 1.44)	.879
3≤ ADLs	0.92 (0.54, 1.57)	.768	0.99 (0.62, 1.57)	.955
Number of comorbidities				
No comorbidities	REF		REF	
1–2 comorbidities	0.58 (0.40, 0.84)	.005	0.54 (0.35, 0.83)	.006
3–4 comorbidities	0.47 (0.30, 0.72)	.001	0.54 (0.35, 0.82)	.005
5+ comorbidities	0.36 (0.19, 0.69)	.002	0.4 (0.21, 0.78)	.008
Self-reported health status				
Poor/fair	REF		REF	
Good	0.78 (0.53, 1.17)	.23	0.72 (0.45, 1.13)	.149
Very good	0.88 (0.57, 1.37)	.582	0.76 (0.49, 1.20)	.235
Excellent	0.96 (0.59, 1.57)	.862	0.88 (0.57, 1.35)	.546
Dementia class				
Probable dementia	REF		REF	
Possible dementia	1.17 (0.57, 2.40)	.678	1.78 (0.90, 3.53)	.099
No dementia	0.96 (0.49, 1.86)	.9	1.48 (0.74, 2.95)	.257
Additional health care coverage				
None	REF		REF	
Medigap	0.7 (0.52, 0.94)	.018	0.7 (0.54, 0.93)	.013
Medicaid	0.84 (0.54, 1.29)	.423	0.72 (0.46, 1.11)	.133
Tricare	1.17 (0.70, 1.94)	.546	1.3 (0.80, 2.10)	.284
Depression	1.01 (0.67, 1.53)	.954	1.02 (0.62, 1.70)	.923

*Notes*: ADLs = activities of daily living; CI = confidence interval; HR = hazard ratio. The proportional hazard assumption for hearing loss group was tested and satisfied in both models. Discrete-time models were estimated with robust pooled cloglog models.

With respect to our additional analyses we found that none of the interaction estimates between depression symptoms, transportation barriers, and hearing status were statistically significant. (Supplementary Tables S2 and S3). In addition, the analysis including all participants who were lost to follow-up showed that although the estimated risk for losing one’s USOC was greater for participants with untreated HL than for older adults without HL, their ratio was not statistically different from one (HR: 1.31; 95% CI: 0.93, 1.86; Supplementary Table S4).

## Discussion

In a sample of 2 110 older Medicare beneficiaries (58.9% female, 20.4% Black), individuals with untreated HL assessed at the beginning of the study had an increased risk of losing their USOC over an 8-year period. There was no significant difference in the risk of reporting losing USOC among participants in the treated- and no-HL groups.

Our results are consistent with a cross-sectional study using data from the Medicare Current Beneficiary Survey (16). According to this study, participants who reported hearing trouble had 1.49 (95% CI: 1.03, 2.14) times the odds of lacking a USOC when compared to participants who reported no trouble hearing.

In contrast to our findings, a previous cross-sectional study (11) using data from the 2015 round of the NHATS found that in fully adjusted models, self-reported HL was not associated with having a USOC (odds ratio = 0.94; 95% CI: 0.59, 1.49). However, the difference can be explained by our time-to-event statistical approach and by the way HL was defined in each study. While in our approach we distinguished between treated and untreated HL, the other study ascertained HL according to participants’ self-reported hearing trouble and deafness, regardless of hearing aid use. Moreover, unlike the other study, our analytic sample included individuals who first reported having a usual provider and who eventually might have lost their USOC.

Communication barriers might be a mediator for the association between hearing health and loss of USOC. In the absence of appropriate accommodations, individuals with HL face difficulties with oral communication. Moreover, given the contribution of HL to cognitive fatigue (23) and memory impairments (24), untreated HL might prevent individuals from seeking and/or maintaining a USOC.

Our results suggest that the use of hearing aids might serve as a protective factor against losing a USOC, as adults with treated HL had similar risk of reporting the loss of USOC compared to those without HL. However, please note that hearing aid users are different from nonusers, as users are on average more educated (25,26). In addition, because Medicare does not cover hearing aids, wealthier households might be more likely to be assigned to the treated-HL group, and because both these characteristics (higher income and education) are protective factors in maintaining a USOC (27), the association between treated HL and risk of losing a USOC might be overestimated. Moreover, hearing aid use itself may reflect unmeasured attitudes toward help-seeking behaviors that could equally be associated with maintaining a USOC (28).

The weighted response rate for NHATS during the initial 2011 cycle was 71.3% out of the individuals originally sampled (70.9% for the unweighted sample). During subsequent years, the response rates for the 2011 cohort were 85.3% (2012), 87.4% (2013), 89.9% (2014), 96% (2015), 88.4% (2016), 95.2% (2017), and 96.2% in 2018. Despite the high response rates in later years we understand that there are important differences between participants who stayed in the study and those who were lost to follow-up. In particular, among participants who met our inclusion criteria at baseline, those who stayed community-dwelling for all eight rounds were younger, reported better health status, and had a lower number of chronic health conditions at baseline than those who were lost to follow-up (see Supplementary Table S1).

Moreover, because we restricted our study sample to participants who remained community-dwelling during the 2011–2018 period, excluded individuals included older adults who might have transitioned into residential care (even momentarily). As a result, a limitation of our study is that our results are not necessarily generalizable to the entire Medicare population, but rather to healthier older adults who might still benefit from hearing health services to maintain a USOC.

Another limitation of our study arises from our measurement of HL. Prior studies using objective audiometric data have estimated that about 63% of adults 70 years of age and older experienced HL (four-frequency pure-tone average ≥25 dB) (12). For the case of the current study, and based on self-reported measures of functional hearing at baseline, we identified only 18.4% (7.3% treated and 11.1% untreated) of participants in our analytic sample as having HL. In contrast, 28.8% of excluded individuals were identified as having HL.

By defining HL based on self-report, our research captures the participants’ everyday hearing functioning, which has been associated with their perceptions regarding access of care (29). However, given the numbers from above, it is clear that we are underestimating the prevalence of untreated HL in our sample. As sensory loss is likely to be underestimated by older adults and those with lower educational attainment (30), our findings might be underestimated by older adults with fewer years of education who are experiencing hearing troubles but who, due to data limitations, were categorized as having no HL. With the recent availability of audiometric data in NHATS, future research should study the association between HL and presence of a USOC.

In sensitivity analyses including individuals who were lost to follow-up during the study period, we found that although the estimated risk for losing one’s USOC was higher for older adults with untreated HL than for those without HL, the CI for the difference still included the null hypothesis (95% CI: 0.93, 1.86). A large proportion of older adults with HL assigned to the no-HL group might underestimate the association and explain the difference with our main findings, particularly if older adults with HL are more likely to be lost to follow-up. It might also be the case that the association between untreated HL and lack of a USOC is moderated by an older adult’s well-being, as those who were observed living in the community during the study period were on average healthier than those lost to follow-up. Additional research exploring the moderation of health status on the association between HL and lack of a USOC is granted.

It is also important to mention that because of the wording of the question pertaining to USOC (“there is a *doctor* you think of …”), some participants whose usual provider is not a physician (eg, nurse practitioner or physician assistant) might respond negatively to this question and thus be coded as lacking a USOC. Unfortunately, due to data availability, we are not able to account for different types of health care providers. While this situation might overestimate the number of study participants without a USOC, we have no reason to assume that people with HL (treated or untreated) might respond differently to this question than older adults without HL.

In addition, given the nature of our data, we did not observe any provider changes. Therefore, our results might be best interpreted in terms of service availability rather than as an ongoing patient–provider relationship. Additionally, our data did not contain information about how participants used their hearing aids (ie, how frequently they used their device). Future research could examine the association between frequency of hearing aid use and having a USOC. Furthermore, additional work (qualitative and quantitative) is needed to better understand the role of communication barriers in an individual’s ability and/or willingness to maintain a USOC.

## Conclusion

Our research shows that HL at baseline is associated with a higher risk of losing one’s USOC during the study period, while highlighting the potential benefits of hearing aids in maintaining one’s USOC. While caution in interpretation is warranted, these results could lend support to policies targeting Americans with HL, such as Medicare coverage of hearing aids, as a means of improving overall health care system use and health outcomes associated with maintaining a USOC.

## Supplementary Material

igad002_suppl_Supplementary_Material
