# Supplementary material for: The Association Between Self-Reported Hearing Loss and Loss of Usual Source of Health Care Among Older Medicare Beneficiaries: Evidence From the National Health and Aging Trends Study
Source: Innov Aging. 2023 Jan 16;7(2):igad002. doi: 10.1093/geroni/igad002 (PMC9999675; doi:10.1093/geroni/igad002)
Supplement: igad002_suppl_Supplementary_Material [file igad002_suppl_supplementary_material.docx]

*Innovation in Aging* Online Supplementary Material: Emmanuel Garcia Morales, Lama Assi, Danielle Powell, Kayti Luu, & Nicholas Reed. Association between self-report hearing loss and loss of usual source of health care among older Medicare beneficiaries: Evidence from the National Health and Aging Trends Study.

**Table S1. Baseline Characteristics of participants excluded and included in our study sample.**

| Characteristic | Total | Excluded Sample | Analytic Sample | p-value |
| --- | --- | --- | --- | --- |
|  | N=7,219 | n=5,109 | n=2,110 |  |
| **Age (years)**, mean (SD) | 77.7 (7.9) | 79.1 (8.0) | 74.5 (6.3) | <0.001 |
| **Female**, n(%) | 4,242 (58.8%) | 2,999 (58.7%) | 1,243 (58.9%) | 0.87 |
| **Race\Ethnicity**, n(%) |  |  |  | <0.001 |
| White | 4,940 (68.4%) | 3,426 (67.1%) | 1,514 (71.8%) |  |
| Black | 1,559 (21.6%) | 1,129 (22.1%) | 430 (20.4%) |  |
| Hispanic\Other | 720 (10.0%) | 554 (10.8%) | 166 (7.9%) |  |
| **Married**, n(%) | 3,627 (50.3%) | 2,404 (47.1%) | 1,223 (58.0%) | <0.001 |
| **Education Level**, n(%) |  |  |  | <0.001 |
| Less than High School | 1,923 (26.6%) | 1,487 (29.1%) | 436 (20.7%) |  |
| High School Diploma or Equivalent | 1,961 (27.2%) | 1,422 (27.8%) | 539 (25.5%) |  |
| Some College or more | 3,249 (45.0%) | 2,114 (41.4%) | 1,135 (53.8%) |  |
| **Household Income**, n(%) |  |  |  | <0.001 |
| Under Poverty Line | 1,646 (22.8%) | 1,290 (25.2%) | 356 (16.9%) |  |
| 100%-199% Poverty Line | 1,954 (27.1%) | 1,460 (28.6%) | 494 (23.4%) |  |
| 200%< Poverty Line | 3,619 (50.1%) | 2,359 (46.2%) | 1,260 (59.7%) |  |
| **Num Comorbidities**, n(%) |  |  |  | <0.001 |
| No comorbidities | 536 (7.4%) | 337 (6.6%) | 199 (9.4%) |  |
| 1-2 Comorbidities | 3,044 (42.2%) | 2,043 (40.0%) | 1,001 (47.4%) |  |
| 3-4 Comorbidities | 2,707 (37.5%) | 1,977 (38.7%) | 730 (34.6%) |  |
| 5+ Comorbidities | 932 (12.9%) | 752 (14.7%) | 180 (8.5%) |  |
| **Self-Reported Health Status**, n(%) |  |  |  | <0.001 |
| Poor\Fair | 2,110 (29.2%) | 1,716 (33.6%) | 394 (18.7%) |  |
| Good | 2,318 (32.1%) | 1,639 (32.1%) | 679 (32.2%) |  |
| Very Good | 1,927 (26.7%) | 1,235 (24.2%) | 692 (32.8%) |  |
| Excellent | 858 (11.9%) | 513 (10.0%) | 345 (16.4%) |  |
| **Dementia Class**, n(%) |  |  |  | <0.001 |
| Probable dementia | 972 (13.5%) | 901 (17.6%) | 71 (3.4%) |  |
| Possible dementia | 938 (13.0%) | 753 (14.7%) | 185 (8.8%) |  |
| No dementia | 5,309 (73.5%) | 3,455 (67.6%) | 1,854 (87.9%) |  |
| **Additional Health Care Coverage,** n(%) |  |  |  | <0.001 |
| None | 2,102 (29.1%) | 1,470 (28.8%) | 632 (30.0%) |  |
| Medigap | 3,533 (48.9%) | 2,418 (47.3%) | 1,115 (52.8%) |  |
| Medicaid | 1,087 (15.1%) | 849 (16.6%) | 238 (11.3%) |  |
| Tricare | 393 (5.4%) | 268 (5.2%) | 125 (5.9%) |  |
| **Depression**, n (%) | 1,153 (16.1%) | 926 (18.4%) | 227 (10.8%) | <0.001 |

**Table S2. Discrete Time Proportional Hazard Models for Losing one’s Usual Source of Care and Hearing Loss at Baseline Including and Interaction Term between Hearing and Depression (n=2,102)**

| Variable | Unweighted | | Weighted | |
| --- | --- | --- | --- | --- |
|  | HR (95% CI) | P-value | HR (95% CI) | P-value |
| **Hearing** |  |  |  |  |
| No Hearing Loss | REF |  | REF |  |
| Hearing Aid Use | 1.27 (0.76,2.14) | 0.362 | 1.07 (0.63,1.83) | 0.795 |
| Untreated Hearing Loss | 1.69 (1.14,2.49) | 0.008 | 1.77 (1.10,2.85) | 0.019 |
| **Depression** | 1.2 (0.77,1.86) | 0.415 | 1.22 (0.71,2.13) | 0.465 |
| **Hearing x Depression** |  |  |  |  |
| Hearing Aid Use x Depression | * |  | * |  |
| Untreated Hearing Loss x Depression | 0.52 (0.18,1.46) | 0.212 | 0.51 (0.18,1.47) | 0.207 |

Notes. HR = hazard ratio. Discrete-time models were estimated with robust pooled cloglog models.

* A total of eight participants who reported using a hearing aid and depression were excluded from the analysis due to small sample size.

**Table S3. Discrete Time Proportional Hazard Models for Losing One’s Usual Source of Care and Hearing Loss at Baseline. Including and Interaction Term between Hearing and Transportation Barriers (n=301^a^)**

| Variable | Unweighted | | Weighted | |
| --- | --- | --- | --- | --- |
|  | HR (95% CI) | P-value | HR (95% CI) | P-value |
| **Hearing** |  |  |  |  |
| No Hearing Loss | REF |  | REF |  |
| Hearing Aid Use | 1.57 (0.31,8.00) | 0.585 | 1.27 (0.14,11.20) | 0.828 |
| Untreated Hearing Loss | 1.86 (0.83,4.16) | 0.13 | 2.04 (0.81,5.14) | 0.127 |
| **Transportation Barrier** | 0.51 (0.21,1.23) | 0.135 | 0.52 (0.18,1.47) | 0.211 |
| **Hearing x Transportation Barrier** |  |  |  |  |
| HA Use x Transportation Barrier | - |  | - |  |
| Untreated Hearing Loss x Transportation Barrier | 0.32 (0.03,3.10) | 0.323 | 0.32 (0.03,3.10) | 0.323 |

Notes. HR = hazard ratio. Discrete-time models were estimated with robust pooled cloglog models.

^a^ A total of eight participants who reported using a hearing aid and transportation barriers, and one who reporting additional health coverage Tricare were excluded from the analysis due to small sample size.

**Table S4.** **Discrete Time Proportional Hazard Models for Losing one’s Usual Source of Care and Hearing Loss at Baseline. Analyses Including Participants Who Were Lost to Follow-Up and Treating them as Right Censored (n=6,304)**

|  | Unweighted | | Weighted | |
| --- | --- | --- | --- | --- |
|  | HR (95% CI) | P-value | HR (95% CI) | P-value |
| **Hearing** |  |  |  |  |
| No Hearing Loss | REF |  | REF |  |
| Hearing Aid Use | 1.29 (0.93,1.79) | 0.127 | 1.1 (0.76,1.60) | 0.602 |
| Untreated Hearing Loss | 1.23 (0.94,1.60) | 0.128 | 1.31 (0.93,1.86) | 0.119 |

Notes. HR = hazard ratio. The proportional hazard assumption for hearing loss group was tested and satisfied in both models. Discrete-time models were estimated with robust pooled cloglog models.
